# Supplementary material for: Supercoiling and looping promote DNA base accessibility and coordination among distant sites
Source: Nat Commun. 2021 Sep 28;12:5683. doi: 10.1038/s41467-021-25936-2 (PMC8478907; doi:10.1038/s41467-021-25936-2)
Supplement: Supplementary file 1 — Supplementary Information [file 41467_2021_25936_MOESM1_ESM.pdf]

Supercoiling and looping promote DNA base accessibility and coordination among distant sites.

Jonathan M. Fogg, Allison K. Judge, Erik Stricker, Hilda L. Chan, and Lynn Zechiedrich.

Supplementary Information:

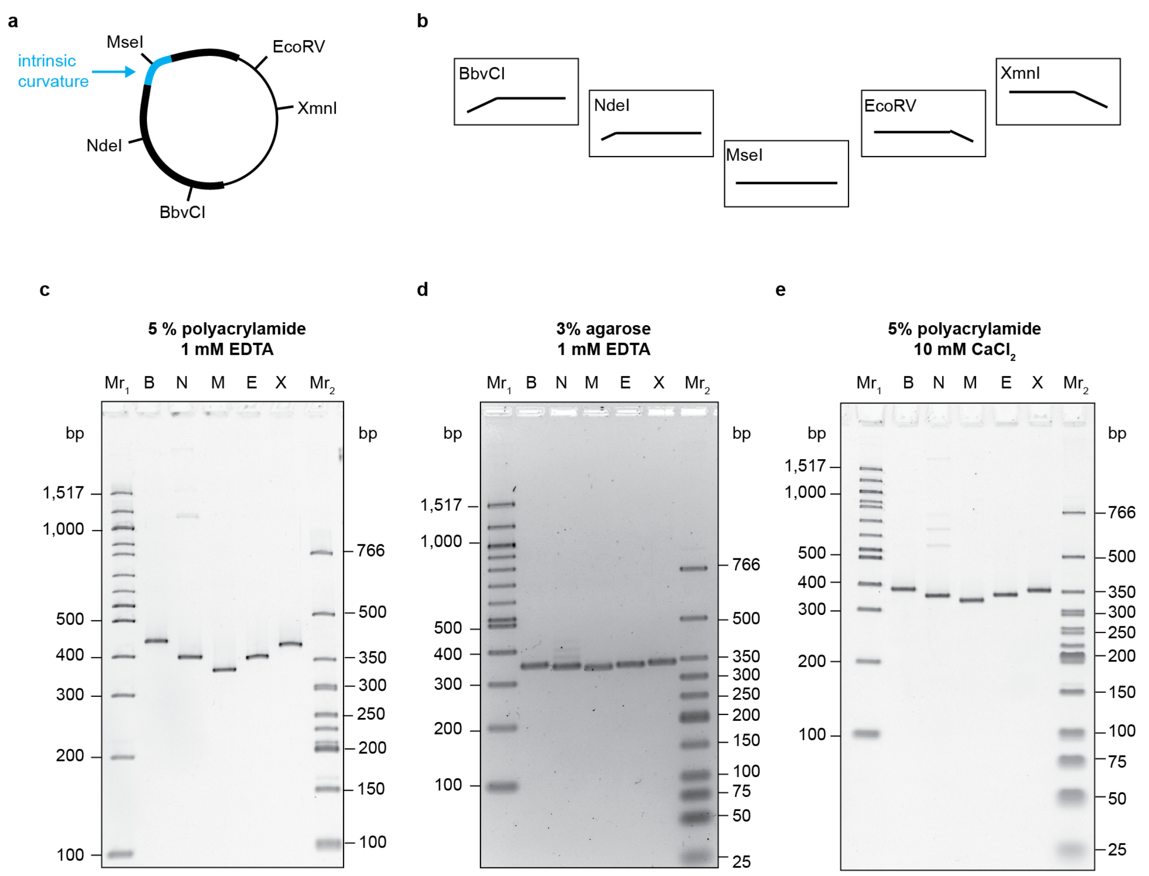

f

Enzyme	PAGE (EDTA)	Agarose (EDTA)	PAGE ($CaCl_2$)
**BbvCI**	403 bp (120 %)	331 bp (99 %)	359 bp (107 %)
**NdeI**	358 bp (120 %)	329 bp (98 %)	341 bp (101 %)
**MseI**	329 bp (98 %)	328 bp (98 %)	330 bp (98 %)
**EcoRV**	358 bp (107 %)	334 bp (99 %)	343 bp (102 %)
**XmnI**	392 bp (117 %)	341 bp (101 %)	354 bp (105 %)

Supplementary Figure 1. Migration of linearized minicircle on polyacrylamide gels varies depending upon the location of the cleavage site. 336 bp minicircle was cleaved with the various restriction enzymes, as indicated, and the products were analyzed by gel electrophoresis. Intrinsic curvature reduces DNA mobility on polyacrylamide gels but does not significantly affect the mobility on agarose gels. (**a**) Map of the 336 bp minicircle sequence showing the positions of the restriction enzymes used. Intrinsic curvature is centered around the MseI site. (**b**) Cleaving the DNA with different restriction enzymes

places the bend at different distances from the end of the molecule. The further the bend is from the ends of the molecule, the slower the migration. (**c**) Polyacrylamide gel electrophoresis in the presence of 1 mM EDTA. (**d**) Agarose gel electrophoresis in the presence of 1 mM EDTA. (**e**) Polyacrylamide gel electrophoresis in the presence of 10 mM $CaCl_2$. Mr_1: 100 bp DNA ladder; B: minicircle cleaved by BbvCI; N: minicircle cleaved by NdeI; M: minicircle cleaved by MseI; E, minicircle cleaved by EcoRV; X minicircle cleaved by XmnI. Mr_2: low molecular weight DNA ladder. (**f**) To quantify the extent to which migration is retarded, apparent DNA lengths were determined using the molecular size calibration feature of ImageQuant TL, with the low molecular weight DNA ladder as the standard. Slower migration leads to a higher apparent DNA length. The values in parentheses show the apparent DNA length as a percentage of the actual DNA length (336 bp). This assay was performed twice with very similar results for each replicate.

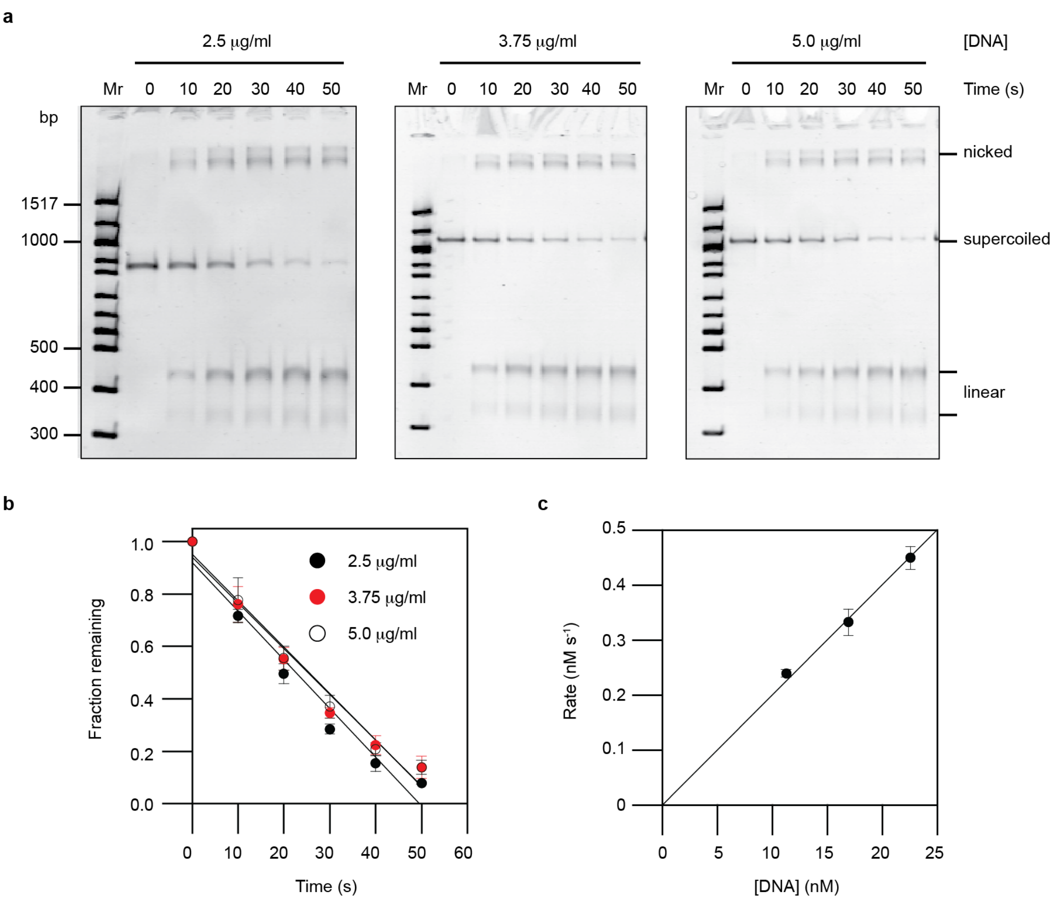

Supplementary Figure 2. Bal-31 is a Very Sensitive Probe of DNA Base Exposure.

(a) 336 bp minicircle DNA (Lk = 26; ΔLk = -6; σ = -0.189) at the three different DNA concentrations indicated, was incubated with a fixed amount of Bal-31 and at the times indicated, samples were removed, quenched by addition of stop buffer, and analyzed by polyacrylamide gel electrophoresis. **(b)** The fraction of supercoiled DNA remaining over time was quantified. The assay was repeated three times for each DNA concentration and the mean values are shown. Error bars show standard deviations. The disappearance of supercoiled substrate over time was fit to linear slopes. (**c**) Reaction rates plotted in terms of moles DNA per unit time (instead of fraction of initial substrate over time) as a function of DNA concentration. Rates were measured three times for each DNA concentration and the mean values are shown. Error bars show standard deviations. Data fitted to a linear slope, passing through the origin, indicating that the rate is proportional to DNA (substrate)

concentration. Assuming the reaction obeys Michaelis-Menten kinetics, if the rate (v) is directly proportional to the substrate concentration ([S]), K_M + [S] must be approximately equal to K_M, such that $v = v_{max}[S]/(K_M+[S])$ simplifies to $v = v_{max}[S]/K_M$ (where v_{max} represents the maximum rate of the enzyme, achieved at saturating substrate concentration). This formula holds true if the K_M value is much higher than the substrate concentrations employed in the assay. Although this analysis was only performed for the $Lk = 26$ ($\Delta Lk = -6$; $\sigma = -0.189$) topoisomer, we assume the findings hold true for other topoisomers.

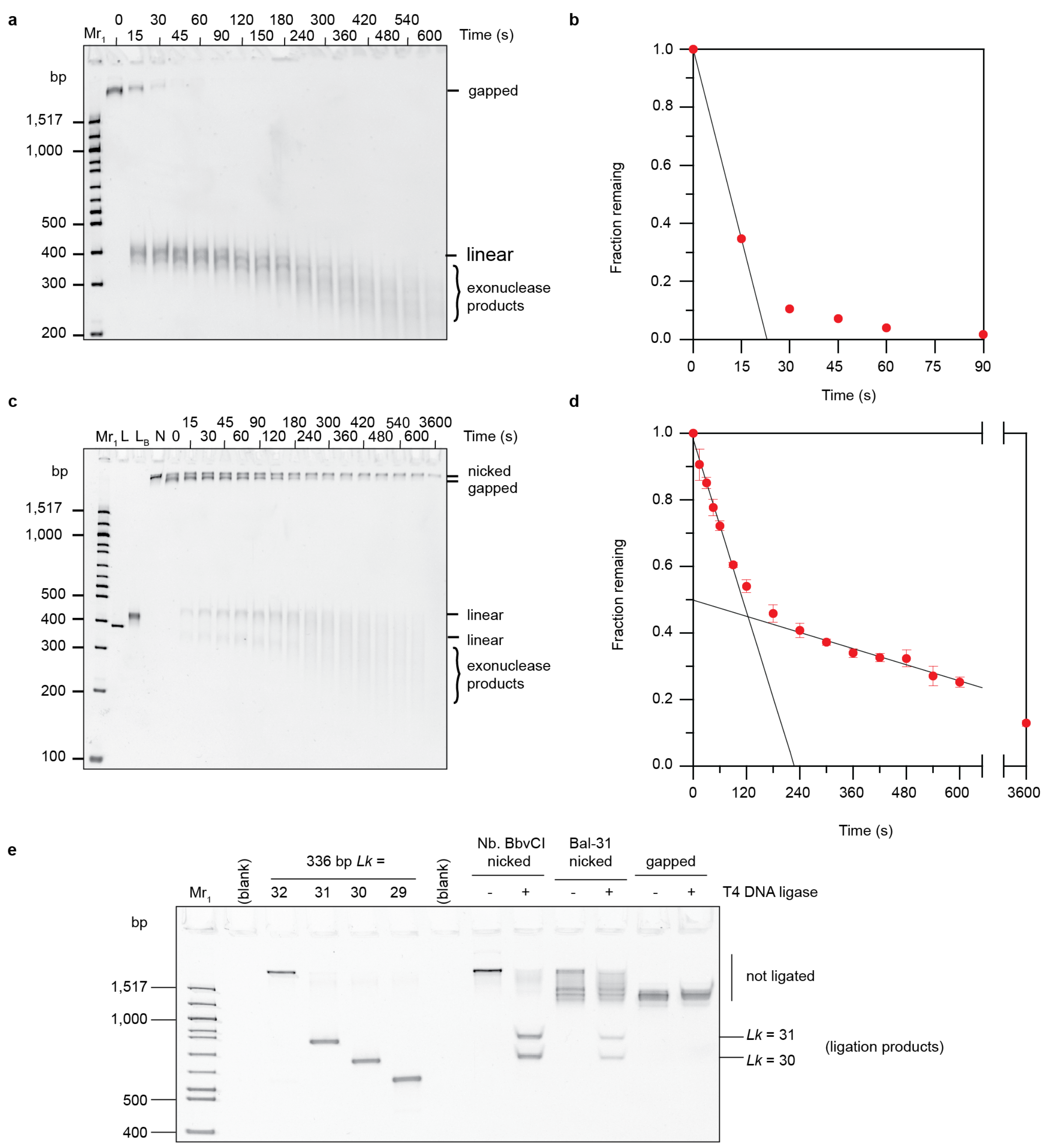

Supplementary Figure 3. Bal-31 preferentially cleaves gapped over nicked DNA minicircles. (**a**) Exonuclease III-gapped minicircle DNA was incubated with Bal-31. At the times indicated, samples were removed, quenched by addition of stop buffer, and analyzed by polyacrylamide gel electrophoresis. Exonuclease III-generated gapped minicircles were cleaved very rapidly by Bal-31 to generate full-length linear DNA. Mr_1: 100 bp DNA ladder. The fraction of gapped DNA remaining over time was quantified and plotted in (**b**). This experiment was performed once. (**c**), Bal-31 cleavage of the strand opposite to the initial

nicking and gapping. Bal-31- nicked and gapped intermediate was isolated by preparative gel electrophoresis and incubated with fresh Bal-31 (time = 0). At the times indicated, samples were removed, quenched by the addition of stop buffer, and analyzed by polyacrylamide gel electrophoresis. Mr_1: 100 bp DNA ladder; L: linear 336 bp DNA (minicircle cleaved by EcoRV); L_B: 336 bp DNA linearized by Bal-31 and gel-purified; N: nicked (336 bp minicircle nicked with Nb.BbvCI). The initial Bal-31 linear products, resulting from cleavage of the second strand, migrate anomalously slowly because of intrinsic curvature in the λ-integrase site, *att*R. With Bal31 re-incubation, linear products were rapidly formed from the Bal31-mediated gapped species, indicating cleavage on the strand opposite to the initial strand cleavage. The presence of a gap accelerated this second-strand cleavage. (d**)** The fraction of Bal-31-nicked and gapped intermediate remaining over time was quantified. The assay was repeated three times and mean values are shown. Error bars represent standard deviations; when not visible, they are smaller than the symbol. The earlier and later regions of the data were fitted to two different linear slopes with the lines extrapolated to the axis limits to facilitate comparison. The multiphasic degradation kinetics reveal that the Bal-31 cleavage rate was approximately 10-fold faster in the first two minutes than at later timepoints. Variability in gap lengths likely explains the different rates of Bal31 cleavage, with larger gaps being cleaved faster. A small fraction (13%) of the Bal-31-nicked intermediate remained even after 60 minutes incubation indicating that it is degraded at a much slower rate. We hypothesized that this slowly degraded fraction may correspond to some of the intermediate lacking a gap, likely resulting from Bal-31 dissociating immediately after making only an initial nick. We, therefore, tested for the presence or absence of a gap by incubating the intermediate with T4 DNA ligase. (**e**) Nb.BbvCI-nicked; Bal-31-nicked; or exonuclease III-gapped DNA were incubated with or without T4 DNA ligase, as indicated, and analyzed by polyacrylamide gel electrophoresis in the presence of 10 mM $CaCl_2$. In

comparison to the exonuclease-gapped circles that migrate as a single band on the gel, the Bal-31-nicked and gapped intermediate has much more heterogenous migration, suggesting a mixture of gap sizes. A small fraction of the intermediate had similar mobility to the Nb.BbvCI species, presumably corresponding to Bal-31 nicked DNA with no gap. Ethidium bromide (1 μg/ml) was included in the ligations to ensure that the ligated products are readily separated from nicked and gapped DNA on the gel. Successful ligation generated *Lk* = 30 and 29 topoisomers as observed with the Nb.BbvCI-nicked control. 336 bp minicircle topoisomer markers (*Lk* = 32, 31, 30, and 29) were also loaded on the gel for comparison. Mr_1: 100 bp DNA ladder. T4 DNA ligase is very inefficient at ligating across a gap. The Nb.BbvCI-nicked control was efficiently repaired by T4 DNA ligase, whereas the gapped control could not be repaired. Of the Bal-31-nicked and gapped intermediate, only ~17% could be repaired by T4 DNA ligase, and thus lacks a gap. The remainder could not be repaired by T4 DNA ligase, confirming that the majority of the Bal-31 intermediate is gapped; thus, resulting in accelerated cleavage by Bal-31. The ligation assay was performed one time.

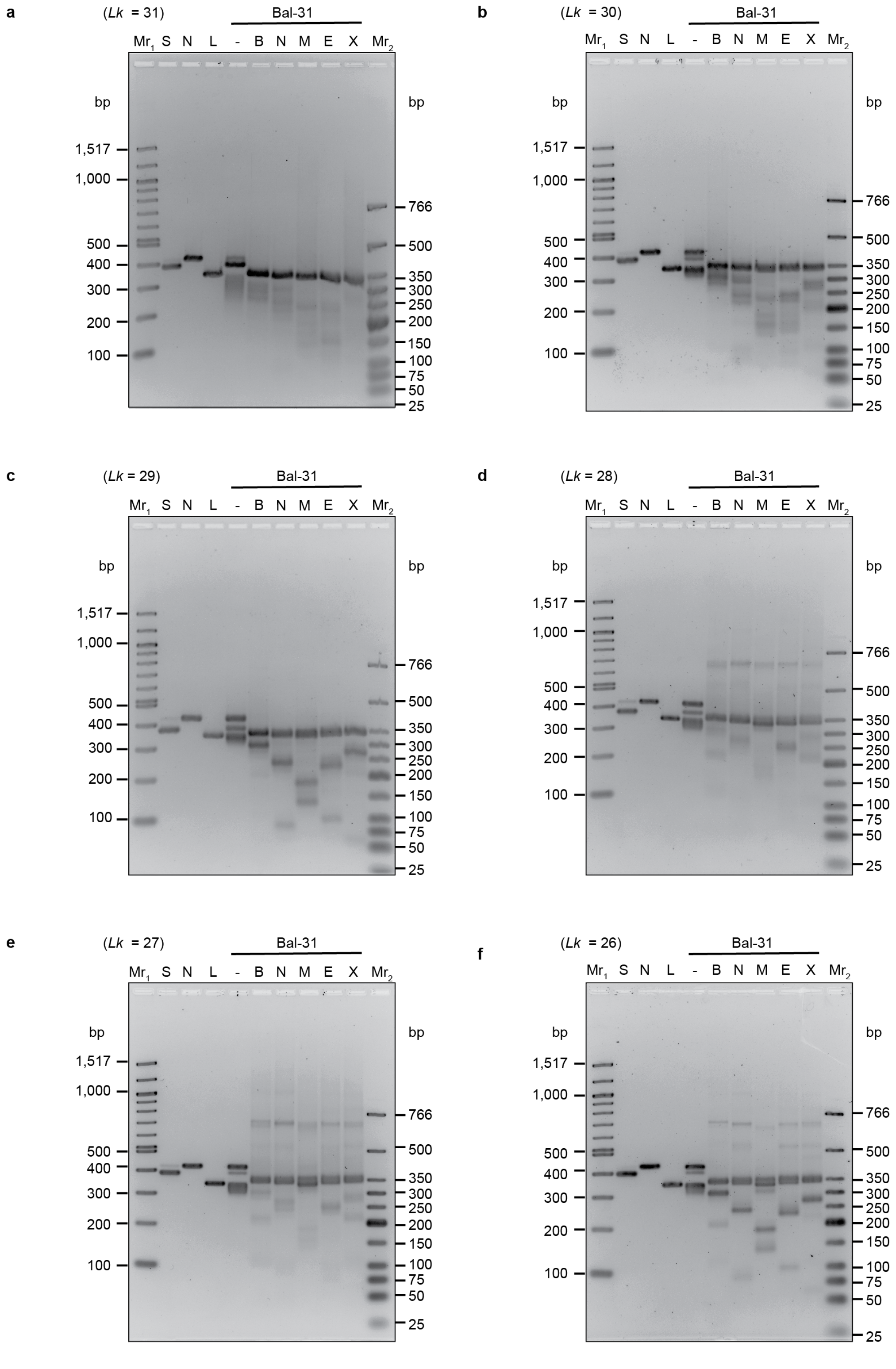

Supplementary Figure 4. Sites of Bal-31 cleavage sites vary with supercoiling.

336 bp minicircle DNA was cleaved with Bal-31, deproteinized, then subsequently cleaved with various restriction enzymes and products separated by agarose gel electrophoresis. **(a)**

Lk = 31 (Δ*Lk* = -1; σ = -0.033). **(b)** *Lk* = 30 (Δ*Lk* = -2; σ = -0.064). **(c)** *Lk* = 29 (Δ*Lk* = -3; σ = -0.095). **(d)** *Lk* = 28 (Δ*Lk* = -4; σ = -0.127). **(e)** *Lk* = 27 (Δ*Lk* = -5; σ = -0.158). **(f)** *Lk* = 26 (Δ*Lk* = -6; σ = -0.189). The lane loading order was the same for each gel. Mr_1: 100 bp DNA ladder; S: supercoiled (uncleaved DNA), N: 336 bp minicircle nicked by Nb.BbvCI; L: 336 bp minicircle linearized by EcoRV; -: minicircle incubated with Bal-31 for 1 minute (10 minutes for *Lk* =31); B, N, M, E, X: minicircle incubated with Bal-31 followed by a second restriction enzyme as indicated (B: BbvCI; N: NdeI; M: MseI; E: EcoRV; X: XmnI); Mr_2: low molecular weight DNA ladder. This assay was performed at least twice for each topoisomer, except for the *Lk* = 30 topoisomer that was assayed just one time. Results were very similar between replicates.

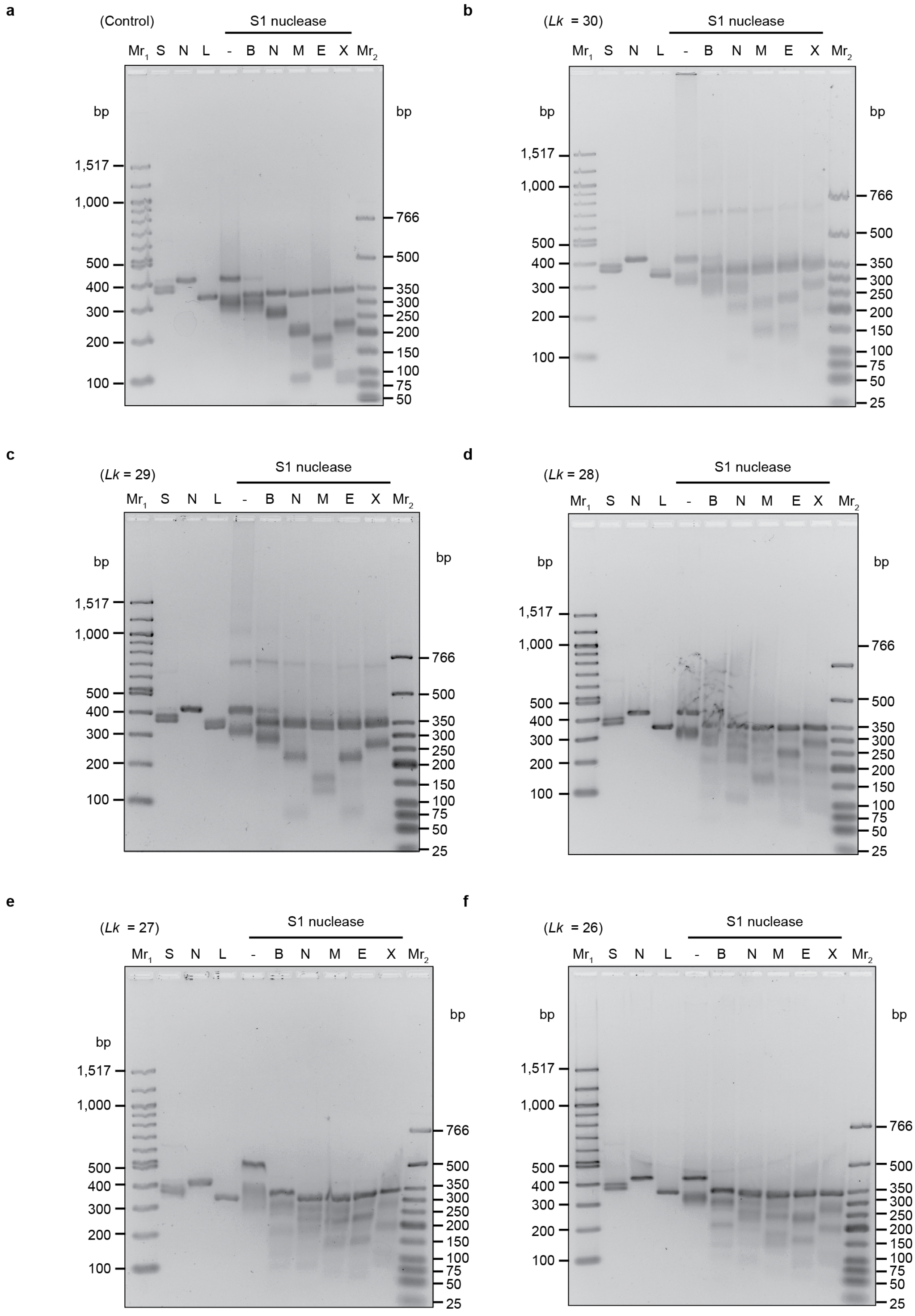

Supplementary Figure 5. Sites of S1 nuclease cleavage sites vary with supercoiling. 336 bp minicircle DNA was nicked by S1 nuclease, followed by a subsequent incubation at a higher enzyme concentration and temperature to linearize the nicked products. S1 nuclease-

linearized DNA was subsequently deproteinized, cleaved with various restriction enzymes and products separated by agarose gel electrophoresis. (**a**) Control reaction with nicked minicircle (nicked at the BbvCI site) to confirm that S1 nuclease cleaves opposite a pre-existing nick. (**b**) *Lk* = 30 (Δ*Lk* = -2; σ = -0.064). (**c**) *Lk* = 29 (Δ*Lk* = -3; σ = -0.095). (**d**) *Lk* = 28 (Δ*Lk* = -4; σ = -0.127). (**e**) *Lk* = 27 Δ*Lk* = -5; σ = -0.158). (**f**) *Lk* = 26 (Δ*Lk* = -6; σ = -0.189). The lane loading order was the same for each gel. Mr_1: 100 bp DNA ladder; S: supercoiled (uncleaved DNA), N: 336 bp minicircle nicked by Nb.BbvCI; L: minicircle linearized by EcoRV; -: minicircle incubated with S1 nuclease; B,N,M,E,X: minicircle incubated with S1 nuclease followed by a second restriction enzyme as indicated (B: BbvCI; N: NdeI; M: MseI; E: EcoRV; X: XmnI); Mr_2: low molecular weight DNA ladder. The assay was performed once for each different topoisomer.

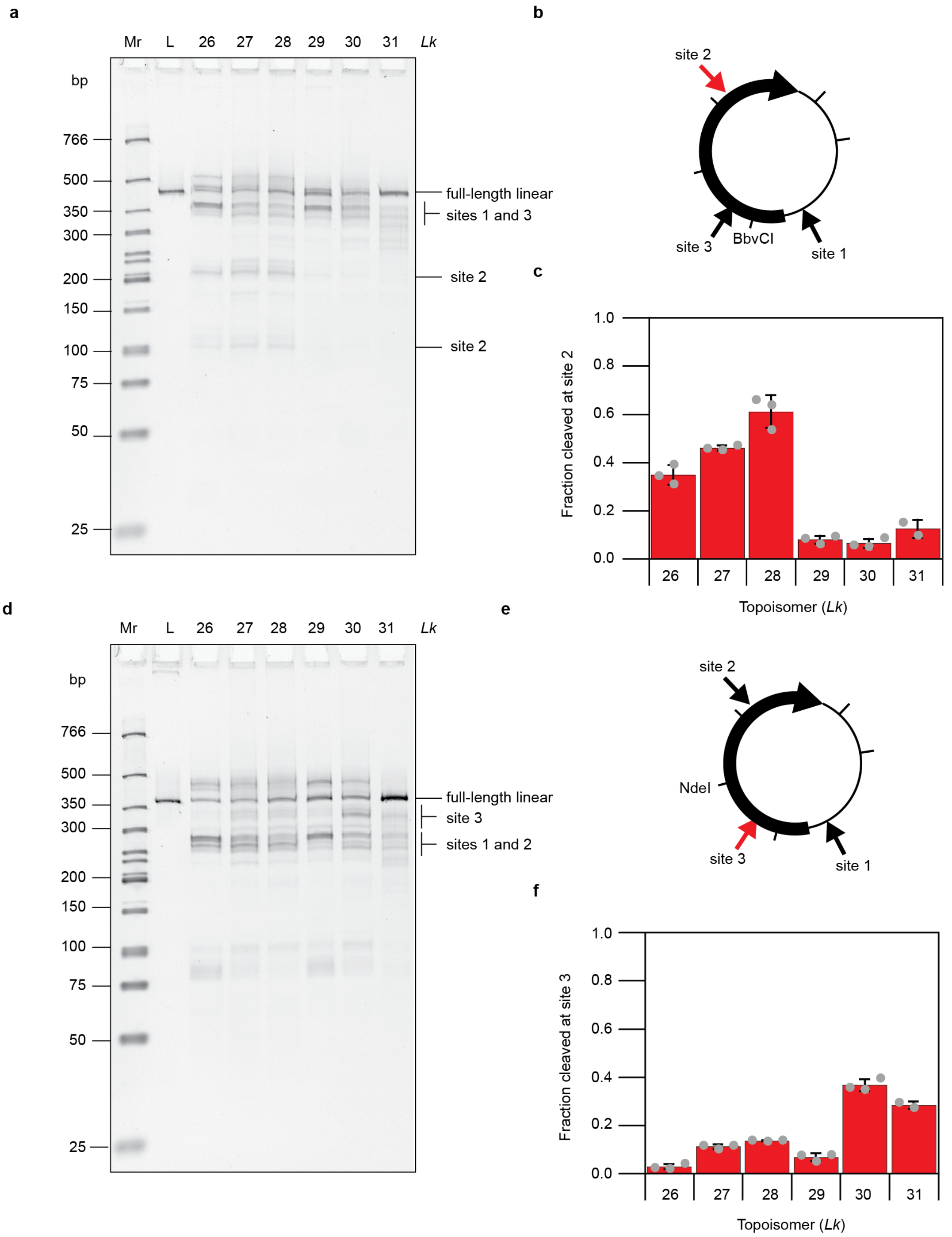

Supplementary Figure 6. Quantitation of site-specific DNA base exposure with supercoiling. Analysis of Bal-31 site preference is shown. A similar analysis was performed for S1 nuclease (see Source Data). Polyacrylamide gels provided more accurate quantitation than agarose gels, because of the sharper bands and reduced background, and were used to determine the relative cleavage at each site. (**a**) 336 bp minicircle DNA with *Lk* as indicated was cleaved with Bal-31, deproteinized, then subsequently cleaved with BbvCI and products

separated by polyacrylamide gel electrophoresis (TAE buffer). Bal-31 cleavage at site 2 results in the smaller fragments when the DNA is subsequently cleaved with BbvCI. The *Lk* = 29 (ΔLk = -3; σ = -0.095) and *Lk* = 26 (ΔLk = -6; σ = -0.189) topoisomers both produced sharp bands. In comparison, the products from the other Bal-31-cleaved topoisomers consist of multiple bands with overlapping migration, suggesting additional minor cleavage site differences as a function of *Lk*. Note that, because of modest intrinsic curvature in the *attR* site, some fragments migrate anomalously slowly on a polyacrylamide gel. Mr: low molecular weight DNA ladder; L: minicircle linearized by BbvCI. The assay was performed three times for each topoisomer with similar results each time (see Source Data). (**b)** Map of the minicircle sequence showing the locations of the three major Bal-31 cleavage sites, and the position of the BbvCI site. Site 2 is highlighted in red. (**c)** Quantitation of the fraction of Bal-31 cleavage occurring at site 2 as a function of *Lk*. Mean values are shown. Error bars show standard deviations. Individual data points for each individual replicate are overlaid as a dot plot. (**d)** 336 bp minicircle DNA with *Lk* as indicated was cleaved with Bal-31, deproteinized, then subsequently cleaved with NdeI and products separated by polyacrylamide gel electrophoresis (TAE buffer). Bal-31 cleavage at site 3 results in larger fragment when the DNA is subsequently cleaved with NdeI. Mr: low molecular weight DNA ladder; L: minicircle linearized by NdeI. The assay was performed three times for each topoisomer with similar results each time (see Source Data). (**e)** Map of the minicircle sequence showing the locations of the three major Bal-31 cleavage sites, and the position of the NdeI site. Site 3 is highlighted in red. **(f)** Quantitation of the fraction of Bal-31 cleavage occurring at site 3 as a function of *Lk*. Mean values are shown. Error bars show standard deviations. Individual data points for each individual replicate are overlaid as a dot plot.

Supplementary Table 1. Details of minicircle topoisomers studied

Minicircle length, bp	*Lk*	Δ*Lk*	Superhelical density (σ)*	Bal-31 cleavage rate (x 10^{-3} s^{-1})	S1 nuclease cleavage rate (x 10^{-3} s^{-1})
333	29	-3	-0.087	12.5 ± 0.3	-
	30	-2	-0.056	13.0 ± 0.7	-
	31	-1	-0.024	0.258 ± 0.044	-
	32	0	+0.007	0.0	-
	33	+1	+0.039	-	-
	34	+2	+0.070	0.0411 ± 0.0099	-
	Nicked		0	0.0623 ± 0.019	-
336	26	-6	-0.189	15.8 ± 0.1	3.85 ± 0.26
	27	-5	-0.158	16.8 ± 0.3	2.47 ± 0.49
	28	-4	-0.127	15.5 ± 0.6	2.11 ± 0.11
	29	-3	-0.095	12.3 ± 0.3	1.50 ± 0.24
	30	-2	-0.064	14.9 ± 0.8	0.284 ± 0.076
	31	-1	-0.033	0.79 ± 0.2	0.0104 ± 0.0033
	32	0	-0.002	0.0	0.0
	33	+1	+0.029	0.0	0.0
	34	+2	+0.060	0.0181 ± 0.0029	0.0
	35	+3	+0.092	0.227 ± 0.013	0.0141 ± 0.0024
	Nicked		0	0.0240 ± 0.0030	-
	Bal-31 nicked		0	4.31[a] ± 0.15<br>0.407[b] ± 0.050	-<br>-
339	29	-3	-0.103	8.15 ± 1.12	-
	30	-2	-0.073	12.9 ± 0.7	-
	31	-1	-0.042	1.61 ± 0.34	-
	32	0	-0.011	0.0	-
	33	+1	+0.020	-	-
	34	+2	+0.051	0.0	-
	35	+3	+0.082	0.0879 ± 0.0166	-
	Nicked		0	0.141 ± 0.005	-
666	56	-8	-0.119	17.3 ± 0.2	-
	57	-7	-0.103	11.6 ± 1.5	-
	58	-6	-0.087	16.9 ± 0.2	-
	59	-5	-0.072	15.8 ± 1.0	-
	60	-4	-0.056	0.659 ± 0.371	-
	61	-3	-0.040	0.958 ± 0.117	-
	62	-2	-0.024	0.0210 ± 0.0023	-
	63	-1	-0.009	0.0121 ± 0.0047	-
	64	0	+0.007	0.00176 ± 0.00305	-
672	57	-7	-0.111	9.54 ± 1.43	-
	58	-6	-0.096	14.9 ± 0.1	-
	59	-5	-0.080	18.1 ± 0.8	-
	60	-4	-0.064	1.59 ± 0.20	-
	61	-3	-0.049	1.65 ± 0.08	-
	62	-2	-0.033	0.0270 ± 0.0011	-
	63	-1	-0.018	0.0174 ± 0.0033	-
	64	0	-0.002	0.00620 ± 0.00320	-

*under Bal-31 reaction conditions (600 mM NaCl, 12 mM $CaCl_2$, 12 mM $MgCl_2$)

[a]initial fast cleavage rate of Bal-31 nicked intermediate (0 to 90s)

[b]subsequent cleavage rate of Bal-31 nicked intermediate for later timepoints (240 to 600s)

Supplementary Table 2. Effect of negative supercoiling on Bal-31 cleavage sites

Topoisomer (336 bp)	Site 1	Site 2	Site 3
$Lk = 26$; $\Delta Lk = -6$; $\sigma = -0.189$	144 (± 6 bp)	311 (± 16 bp)	N.D.
$Lk = 27$; $\Delta Lk = -5$; $\sigma = -0.158$	137 (± 7 bp)	300 (± 15 bp)	N.D.
$Lk = 28$; $\Delta Lk = -4$; $\sigma = -0.127$	149 (± 22 bp)	293 (± 8 bp)	N.D.
$Lk = 29$; $\Delta Lk = -3$; $\sigma = -0.095$	143 (± 8 bp)	305 (± 20 bp)	N.D.
$Lk = 30$; $\Delta Lk = -2$; $\sigma = -0.064$	138 (± 12 bp)	N.D.	200 (± 10 bp)
$Lk = 31$; $\Delta Lk = -1$; $\sigma = -0.033$	145 (± 14 bp)	N.D.	206 (± 8 bp)
Mean across all topoisomers	143 (± 5 bp)	302 (± 8 bp)	203 (± 4 bp)

DNA fragment lengths resulting from minicircle cleavage with Bal-31 subsequently cleaved with restriction enzymes reveal the distance of each Bal-31 cleavage site from the known restriction site (see Methods). Use of multiple different restriction endonucleases allowed the cleavage site to be determined from different directions around the circle. The cleavage site location was determined for each individual fragment length. The locations derived from multiple different fragment lengths from multiple different restriction digests were averaged. Standard deviation of these measurements is shown in bp. Both the mean and standard deviation values are rounded to the nearest base pair. N.D.: not determined because no significant Bal-31 cleavage was observed. Further details including measured fragment lengths are provided in Source Data.

Supplementary Table 3. Effect of negative supercoiling on S1 nuclease cleavage sites

Topoisomer (336 bp)	Site 1	Site 2	Site 3
$Lk = 26$; $\Delta Lk = -6$; $\sigma = -0.189$	142 (± 8 bp)	317 (± 14 bp)	201 (± 1 bp)
$Lk = 27$; $\Delta Lk = -5$; $\sigma = -0.158$	141 (± 18 bp)	310 (± 27 bp)	204 (± 12 bp)
$Lk = 28$; $\Delta Lk = -4$; $\sigma = -0.127$	128 (± 9 bp)	289 (± 6 bp)	215 (± 11 bp)
$Lk = 29$; $\Delta Lk = -3$; $\sigma = -0.095$	141 (± 17 bp)	N.D.	N.D.
$Lk = 30$; $\Delta Lk = -2$; $\sigma = -0.064$	150 (± 20 bp)	N.D.	202 (± 12 bp)
Mean across all topoisomers	140 (± 8 bp)	305 (± 15 bp)	206 (± 6 bp)

Cleavage sites were determined as for Bal-31 (Supplemental Table 2). N.D.: not determined because no significant S1 nuclease cleavage was observed. Further details including measured fragment lengths are provided in Source Data.
