# Supplementary material for: Supercoiling and looping promote DNA base accessibility and coordination among distant sites
Source: Nat Commun. 2021 Sep 28;12:5683. doi: 10.1038/s41467-021-25936-2 (PMC8478907; doi:10.1038/s41467-021-25936-2)
Supplement: Supplementary file 3 — Reporting Summary [file 41467_2021_25936_MOESM3_ESM.pdf]

nature portfolio

Corresponding author(s): Lynn Zechiedrich

Last updated by author(s): Aug 13, 2013

Reporting Summary

Nature Portfolio wishes to improve the reproducibility of the work that we publish. This form provides structure for consistency and transparency in reporting. For further information on Nature Portfolio policies, see our Editorial Policies and the Editorial Policy Checklist.

Statistics

For all statistical analyses, confirm that the following items are present in the figure legend, table legend, main text, or Methods section.

n/a	Confirmed	
☒	☐	The exact sample size (*n*) for each experimental group/condition, given as a discrete number and unit of measurement
☐	☒	A statement on whether measurements were taken from distinct samples or whether the same sample was measured repeatedly
☒	☐	The statistical test(s) used AND whether they are one- or two-sided<br>*Only common tests should be described solely by name; describe more complex techniques in the Methods section.*
☒	☐	A description of all covariates tested
☒	☐	A description of any assumptions or corrections, such as tests of normality and adjustment for multiple comparisons
☐	☒	A full description of the statistical parameters including central tendency (e.g. means) or other basic estimates (e.g. regression coefficient) AND variation (e.g. standard deviation) or associated estimates of uncertainty (e.g. confidence intervals)
☒	☐	For null hypothesis testing, the test statistic (e.g. *F*, *t*, *r*) with confidence intervals, effect sizes, degrees of freedom and *P* value noted<br>*Give P values as exact values whenever suitable.*
☒	☐	For Bayesian analysis, information on the choice of priors and Markov chain Monte Carlo settings
☒	☐	For hierarchical and complex designs, identification of the appropriate level for tests and full reporting of outcomes
☒	☐	Estimates of effect sizes (e.g. Cohen's *d*, Pearson's *r*), indicating how they were calculated

Our web collection on statistics for biologists contains articles on many of the points above.

Software and code

Policy information about availability of computer code

Data collection	PC Image v10.5
Data analysis	ImageQuant TL v8.1, Kaleidagraph v5.1, TotalLab TL100 v 2009.

For manuscripts utilizing custom algorithms or software that are central to the research but not yet described in published literature, software must be made available to editors and reviewers. We strongly encourage code deposition in a community repository (e.g. GitHub). See the Nature Portfolio guidelines for submitting code & software for further information.

Data

Policy information about availability of data

All manuscripts must include a data availability statement. This statement should provide the following information, where applicable:

- Accession codes, unique identifiers, or web links for publicly available datasets
- A description of any restrictions on data availability
- For clinical datasets or third party data, please ensure that the statement adheres to our policy

The published article includes all datasets generated during this study. Gels used to map enzyme cleavage sites are included in Supplementary Figure 4, Supplementary Figure 5, and Supplementary Figure 6. Raw data and uncropped gel images are provided in the supplementary Source Data file. Enzyme cleavage rates used to generate the graphs in Figures 4 and 5 are listed in Supplementary Table 1 and in the Source Data file.

Field-specific reporting

Please select the one below that is the best fit for your research. If you are not sure, read the appropriate sections before making your selection.

☒ Life sciences ☐ Behavioural & social sciences ☐ Ecological, evolutionary & environmental sciences

For a reference copy of the document with all sections, see nature.com/documents/nr-reporting-summary-flat.pdf

Life sciences study design

All studies must disclose on these points even when the disclosure is negative.

Sample size	Not applicable. The relevant parameters (DNA supercoiling and minicircle size) are precisely controlled and do not vary within a given population.
Data exclusions	No data were excluded from analyses
Replication	The number of times each experiment was performed is now reported in the paper. For enzyme kinetics, the experiment was typically performed in triplicate. In most cases the variability between runs was very small. For the cleavage site mapping experiments each experiment was typically performed twice but each experiment mapped the cleavage sites using multiple different enzymes essentially providing up to 5 technical replicates for each cleavage site.
Randomization	Not applicable. The study involves DNA minicircles prepared to homogeneity with well defined levels of supercoiling. Because the major variable (DNA supercoiling) is precisely known, randomization should not be necessary.
Blinding	Not applicable. All the parameters measured are precisely defined (e.g. enzyme cleavage rate) and not subjective. Blinding shoule not be necessary.

Reporting for specific materials, systems and methods

We require information from authors about some types of materials, experimental systems and methods used in many studies. Here, indicate whether each material, system or method listed is relevant to your study. If you are not sure if a list item applies to your research, read the appropriate section before selecting a response.

Materials & experimental systems

n/a	Involved in the study	
☒	☐	Antibodies
☒	☐	Eukaryotic cell lines
☒	☐	Palaeontology and archaeology
☒	☐	Animals and other organisms
☒	☐	Human research participants
☒	☐	Clinical data
☒	☐	Dual use research of concern

Methods

n/a	Involved in the study	
☒	☐	ChIP-seq
☒	☐	Flow cytometry
☒	☐	MRI-based neuroimaging
